# From Weight Bias Internalization to Health-Related Quality of Life: Self-esteem and Psychopathology in Pre-bariatric Surgery Patients

**DOI:** 10.1007/s11695-022-06261-z

**Published:** 2022-09-03

**Authors:** Xu Liu, Wenjing Zhang, Wenwen Yue, Chaonan Sun, Weihua Li

**Affiliations:** 1 Operating Theater, Qilu Hospital, Cheeloo College of Medicine, Shandong University, Jinan, 250012 People’s Republic of China; 2 School of Nursing and Rehabilitation, Cheeloo College of Medicine, Shandong University, Jinan, 250012 People’s Republic of China

**Keywords:** Weight bias internalization, Health-related quality of life, Self-esteem, Psychopathology, Bariatric surgery

## Abstract

**Introduction:**

It has been reported that people seeking bariatric surgery have poor health-related quality of life (HRQoL). Weight bias internalization (WBI) is prevalent in this population and strongly associated with psychopathology and health status. However, the psychological mechanisms underlying the relationship between WBI and the physical and mental dimensions of HRQoL remain to be clarified.

**Methods:**

A preoperative sample of patients with obesity (*N* = 246; women = 75.2%; *M*_age_ = 32.07) completed validated measures as part of a routine preoperative psychological assessment to assess their WBI, self-esteem, anxiety symptoms, depressive symptoms, and HRQoL.

**Results:**

After controlling for the effects of gender, age, and BMI, WBI was linked to poorer physical and mental HRQoL through low self-esteem and increased psychological distress, including anxiety and depressive symptoms.

**Conclusion:**

In pre-bariatric surgery patients with obesity, high WBI may predict impairments in mental and physical HRQoL by lowering self-esteem, and further increasing anxiety and depressive symptoms. Interventions targeting WBI may be an important aspect to consider in the clinical treatment of pre-bariatric surgery patients. Further longitudinal studies are warranted to determine causality.

**Graphical abstract:**

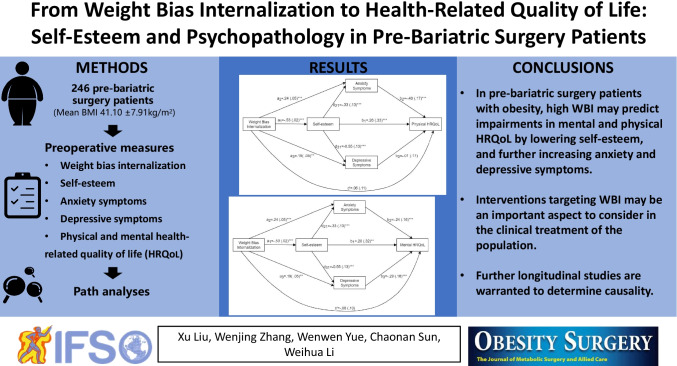

## Introduction

Obesity is a growing global epidemic and bariatric surgery has become an accepted therapy for this condition [1, 2]. Research suggests that concerns regarding quality of life (QoL) have increased among populations with obesity [3]. Health-related quality of life (HRQoL) is used as a subjective multidimensional assessment index of physical and psychological status [4]. Patients with obesity have been shown to have poorer HRQoL in multiple domains [5]. Damage to HRQoL is usually attributed to the physical health damage caused by weight gain. However, research has shown that widespread social bias or discrimination against people with obesity and related psychopathological features may harm their physiological and psychological health [6].

In various social settings, people with obesity are generally susceptible to weight bias, specifically referring to weight-related stereotypes such as laziness, incompetence, stupidity, and lack of willpower [7]. It is worth noting that one of the most detrimental sides of this stigma is its tendency to be internalized by those subjected to it. Owing to social identity issues, these negative weight stereotypes and societal devaluations are further internalized into self-disparagement, which is also called weight bias internalization (WBI) [8]. It has been reported that the contribution of WBI to variance in psychopathology goes beyond that of general stigmatizing attitudes [9]. Studies have also confirmed that negative effects associated with WBI can impair health outcomes and QoL [10–12]. However, the effect of WBI on the HRQoL of patients with obesity remains under exploration. Further investigations are needed on the underlying psychological mechanisms of the association between WBI and HRQoL in patients seeking bariatric surgery for obesity treatment, which is arguably one of the most relevant samples within this context. Research has shown that individuals who undergo bariatric surgery report particularly high levels of WBI [13]. Furthermore, a systematic review of obesity concluded that people seeking bariatric surgery had the worst HRQoL [14]. Thus, investigating the mechanisms underlying the correlation between WBI and HRQoL in pre-bariatric surgery patients is especially significant and could inform surgical treatment of obesity.

Self-esteem, defined as the intrinsically subjective global self-evaluation of one’s own values [15], and negative psychological states may be potential mediators of the association between WBI and HRQoL. Self-esteem is an essential indicator of positive mental health [16], with individuals with higher self-esteem showing clearer self-cognition in the cited study. In contrast, WBI and negative self-beliefs have been linked to lower self-esteem [17]. Due to the strong correlation between stigma and psychopathology, several studies have demonstrated a negative effect of WBI on psychological well-being [8, 10, 18, 19]. To the best of our knowledge, however, few studies have examined whether WBI further affects the overall health status of patients with obesity by lowering their self-esteem and influencing psychological states.

According to the transdiagnostic theory [20], psychological distress may trigger dysfunctional mood modulatory behaviors, such as binge eating or self-injury. Thus, one of the mechanisms underlying these negative health outcomes may be the induction of maladaptive emotional states. Another significant question that has not been fully resolved in previous studies is whether WBI is associated with different mental health statuses. Anxiety is one of the most commonly reported negative emotions in people with obesity, and although its incidence is similar to that of depression in this population, the conceptualization of these two constructs differs [21]. However, few studies have examined the effects of anxiety on the association between WBI and HRQoL in patients seeking bariatric surgery; and inconsistent findings exist regarding the correlation between WBI and anxiety symptoms [6]. Therefore, it is imperative to further explore these topics.

Although WBI has been demonstrated to negatively impact both physical and psychological health [6], the psychological mechanisms underlying the relationship between WBI and QoL in different domains may not be consistent. Additionally, most studies have focused on the psychological outcomes associated with WBI or perceived weight stigma [19]. To identify the possible differential effect of WBI on individual physical and mental health, the current study explored the psychological mechanisms of the association between WBI and HRQoL in physical and mental domains in pre-bariatric surgery patients, respectively.

## Materials and Methods

### Participants and Procedure

Candidates for laparoscopic sleeve gastrectomy were recruited from the Department of General Surgery of Qilu Hospital of Shandong University, between December 2020 and September 2021. All patients who met the criteria for bariatric surgery and were able to complete the questionnaires independently were eligible to participate. The surgical criteria were (1) being aged between 16 and 65 years, and (2) having a body mass index (BMI) ≥ 32.5 kg/m^2^ or between 27.5 and 32.5 kg/m^2^ with comorbidities that were difficult to control through lifestyle changes and medical treatment. The patients completed the questionnaires at the clinic as part of a routine preoperative psychological assessment, and then were invited to participate in the study. Verbal informed consent was obtained from all the participants. The study was approved by the Institutional Review Board of the School of Nursing and Rehabilitation of Shandong University. Of the 255 candidates confirmed eligible by prescreening, 252 completed the questionnaire, of which 246 consented, and were eventually enrolled in the study. There were no significant differences in age, gender, and BMI between bariatric surgery candidates who were not included and participants who were enrolled in the study.

### Measures

#### Demographics

Data, including age and gender, were extracted from the candidates’ clinical records. Height, weight, waistline, and hipline were measured on admission by using professional instruments. The average values were obtained from three repeated measurements. BMI was calculated based on weight and height.

#### WBI

Eleven-item Weight Bias Internalization Scale-Modified (WBIS) [9] was used to assess the degree that a person with obesity applies negative weight-based stereotypes. Items were answered using a 7-point rating scale with a range of 1 to 7 (strongly disagree to strongly agree). Higher scores represented higher levels of WBI. The WBIS has demonstrated strong internal consistency [9] and has been previously used in samples of overweight or obese participants [12, 17]. Cronbach’s *α* was 0.845.

#### Self-esteem

Ten-item Rosenberg Self-Esteem Scale (SES) was used to measure participants’ self-esteem [15]. Items were answered using a 4-point scale ranging from 1 to 4 (absolutely right to absolutely wrong). Higher scores indicated higher levels of self-esteem. This scale has been used previously for samples seeking bariatric treatment [17]. Cronbach’s *α* was 0.846.

#### Depressive Symptoms

Depressive symptoms within the past month were screened using the 20-item Self-Rating Depression Scale (SDS), which is a self-report measure widely conducted to identify the presence of depressive symptoms [22]. Items were answered using a 4-point rating scale ranging from 1 to 4 (less time to most of the time). The sum scores of all items were calculated as the total scores (*X*), while the standard scores were calculated using the following formula: *Y* = 1.25*X*, taking the integer part. The symptom severity was classified as mild (53–62), moderate (63–72), or severe (≥ 73). The threshold score for screening depressive symptoms was ≥ 53. The scale has been used in bariatric surgery patients [23] and has been validated in a Chinese sample [24]. Cronbach’s *α* was 0.802.

#### Anxiety Symptoms

Anxiety symptoms within the past month were screened using the 20-item Self-Rating Anxiety Scale (SAS), which is a self-reported measure widely conducted to identify the presence of anxiety symptoms [22]. Items were answered using a 4-point rating scale ranging from 1 to 4 (less time to most of the time). The sum scores of all items were calculated as the total scores (*X*), while the standard scores were calculated using the following formula: *Y* = 1.25*X*, taking the integer part. The symptom severity was classified as mild (50–59), moderate (60–69), or severe (≥ 70). The threshold score for screening anxiety symptoms was ≥ 50. This scale has been used for samples with obesity [25]. Cronbach’s *α* was 0.745.

#### HRQoL

Physical and mental HRQoL was assessed using the 36-item Short-Form Health Survey, which has satisfactory validity in patients with morbid obesity [26]. It comprises eight subfactors: (1) physical function; (2) role-physical; (3) role-emotional; (4) bodily pain; (5) vitality; (6) social function; (7) mental health; (8) general health. The four physical and four mental subfactors were separately categorized into the physical component summary, which assesses physical HRQoL, and the mental component summary, which assesses mental HRQoL. Cronbach’s *α* was 0.809.

### Statistical Analysis

All analyses were performed using SPSS version 25.0. Before testing the hypotheses, we conducted a preliminary analysis. The Shapiro–Wilk test was carried out to check the assumption of a normal distribution. Spearman’s correlation analysis, independent-samples *t*-test, and one-way ANOVA were performed to compare the differences in all scale scores by gender, age, and BMI.

SPSS-Hayes PROCESS version 3.4. was used for two path analyses. WBI was the predictor variable, and physical HRQoL and mental HRQoL were outcome variables, respectively, to test whether the effects of WBI on physical and mental HRQoL were accounted for through indirect effects of self-esteem, anxiety, and depressive symptoms. The percentile bootstrap method (*n* = 5000) was used to calculate the 95% confidence intervals (CIs). This effect was deemed statistically significant if the 95% confidence interval did not include zero. Analyses were performed with statistical significance set at a two-tailed *α* value of 0.05.

## Results

### Preliminary Analyses

A total of 246 participants completed the questionnaires and were enrolled in this study. The study participants (*n* = 246) did not differ significantly from those who did not complete the survey (*n* = 3) or refused to participate (*n* = 6) in terms of age, gender, and BMI (*p*s > 0.05). Table 1 shows the demographic characteristics of the participants and their self-esteem, anxiety symptoms, and depressive symptoms score classifications. The mean age of all participants was 32.07 years (standard deviation (SD) = 7.50) and BMI was 41.10 kg/m^2^ (SD = 7.91). Of these, 61 (24.8%) were male and 185 (75.2%) were female.

**Table 1:** Demographic characteristics and score classifications of the self-esteem, anxiety and depressive symptoms

	Mean ± SD or *N* (%)
Age (years)	32.07 ± 7.50
Gender	
Female	185 (75.2%)
Male	61 (24.8%)
Height (m)	1.68 ± 0.77
Weight (kg)	117.32 ± 27.84
BMI (kg/m^2^)	41.10 ± 7.91
Waistline (cm)	121.57 ± 17.61
Hipline (cm)	128.84 ± 15.57
Waist hip rate	0.94 ± 0.08
Self-esteem	
Normal (20 ~ 30)	153 (62.2%)
Moderate (30 ~ 35)	68 (27.6%)
High (> 35)	16 (6.5%)
Anxiety symptoms	
Mild (50 ~ 59)	59 (24.0%)
Moderate (60 ~ 69)	30 (12.2%)
Severe (≥ 70)	1 (0.4%)
Depressive symptoms	
Mild (53 ~ 62)	65 (26.4%)
Moderate (63 ~ 72)	25 (10.2%)
Severe (≥ 73)	8 (3.3%)

*BMI*, body mass index; *SD*, standard deviation; *N*, number. Age, height, weight, BMI, waistline, hipline, and waist hip rate are expressed as mean ± SD. Other data are numbers (percentages)

The results of the descriptive statistics and Spearman’s correlation analysis for BMI, age, gender, and scale scores are shown in Table 2. The average WBI was 52.02 ± 11.66, indicating high WBI levels among participants. Scores for anxiety and depressive symptoms averaged at 48.48 ± 8.46 and 50.93 ± 10.01, respectively; hence, they were very close to the thresholds of ≥ 50 and ≥ 53. The average scores for self-esteem, physical HRQoL, and mental HRQoL were 28.86 ± 4.27, 64.91 ± 18.61, and 64.15 ± 20.99, respectively, which were relatively high. We standardized the BMI logarithmically because it did not satisfy the normality test. BMI was significantly correlated with physical HRQoL (*p* < 0.01). Age was significantly correlated with each study variable (*ps* < 0.05), except for physical HRQoL. Gender was significantly correlated with WBI, anxiety and depressive symptoms, and mental HRQoL (*p*s < 0.05). Compared with men, women reported greater WBI (*t* =  − 3.47, *p* < 0.01), anxiety symptoms (*t* =  − 2.04, *p* = 0.04), depressive symptoms (*t* =  − 2.95, *p* < 0.01), and poorer mental HRQoL (*t* = 2.21, *p* = 0.03). Consequently, gender, age, and BMI were controlled as covariates in all path analyses. As expected, WBI was negatively correlated with both physical (*r* =  − 0.23, *p* < 0.01) and mental (*r* =  − 0.46, *p* < 0.01) HRQoL.

**Table 2:** Descriptive statistics and correlations for main variables

	1	2	3	4	5	6	7	8	9	Mean	SD
BMI	-										
Age	− 0.21**	-									
Gender	− 0.33**	0.07	-								
WBI	− 0.07	− 0.14*	0.22**	-						52.02	11.66
Self-esteem	− 0.04	0.16*	− 0.12	− 0.54**	-					28.86	4.27
Anxiety symptoms	− 0.06	− 0.19**	0.13*	0.45**	− 0.48**	-				48.48	8.46
Depressive symptoms	− 0.10	− 0.13*	0.19**	0.51**	− 0.66**	0.71**	-			50.93	10.01
Physical HRQoL	− 0.17**	0.01	− 0.05	− 0.23**	0.41**	− 0.46**	− 0.38**	-		64.91	18.61
Mental HRQoL	0.06	0.20**	− 0.14*	− 0.46**	0.56**	− 0.60**	− 0.65**	0.59**	-	64.15	20.99

*WBI*, weight bias internalization; *HRQoL*, health-related quality of life; *SD*, standard deviation. *P < 0.05; **P < 0.01

### Path Analyses

**Fig. 1:**
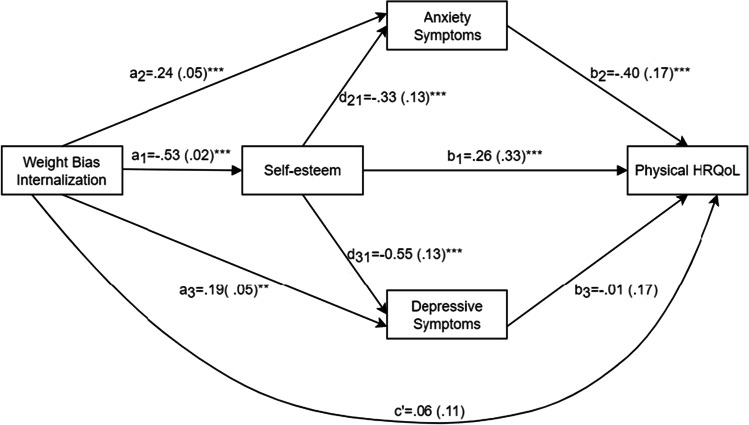
Standardized path coefficients and standard errors of the PROCESS path model among weight bias internalization, self-esteem, anxiety symptoms, depressive symptoms, and physical HRQoL. ***P < 0.001

The standardized path coefficients and standard errors (SEs) of the path model with scores for physical HRQoL as the dependent variable are presented in Fig. 1. Our results showed that all path coefficients were significant, except for the associations between WBI and physical HRQoL, and between depressive symptoms and physical HRQoL. The total effect of WBI on physical HRQoL was significant (*β* =  − 0.39, 95%CI = [− 0.59, − 0.19]). The direct effect of WBI on physical HRQoL was not significant. Table 3 shows the standardized coefficients and CIs for the total and individual indirect effects and the model statistics. The results showed that self-esteem mediated the association between WBI and physical HRQoL (*β* =  − 0.14, 95%CI = [− 0.23, − 0.06]). Anxiety symptoms also mediated this association (*β* =  − 0.09, 95%CI = [− 0.16, − 0.04]). The chain-mediating effect of WBI on physical HRQoL through self-esteem and anxiety symptoms (WBI → self-esteem → anxiety symptoms → physical HRQoL) was also significant.

**Table 3:** Mediating effect of self-esteem, anxiety symptoms, and depressive symptoms in the association between WBI and physical HRQoL, controlling for gender, age, and BMI. Standardized coefficients reported

Effect	*β* (SE)	95%CI	Proportion	*n*	Model *R*^*2*^	*F* (*df*)
Total indirect effect	− 0.30 (0.04)	[− 0.39, − 0.22]	100.00%	246	0.31	15.19 (7,238)***
WBI → self-esteem → physical HRQoL	− 0.14 (0.04)	[− 0.23, − 0.06]	46.12%			
WBI → anxiety symptoms → physical HRQoL	− 0.09 (0.03)	[− 0.16, − 0.04]	31.52%			
WBI → depressive symptoms → physical HRQoL	0.002 (0.02)	[− 0.04, 0.04]				
WBI → self-esteem → anxiety symptoms → physical HRQoL	− 0.07 (0.02)	[− 0.12, − 0.03]	23.66%			
WBI → self-esteem → depressive symptoms → physical HRQoL	0.002 (0.03)	[− 0.05, 0.06]				

*WBI*, weight bias internalization; *HRQoL*, health-related quality of life. ***P < 0.001

**Fig. 2:**
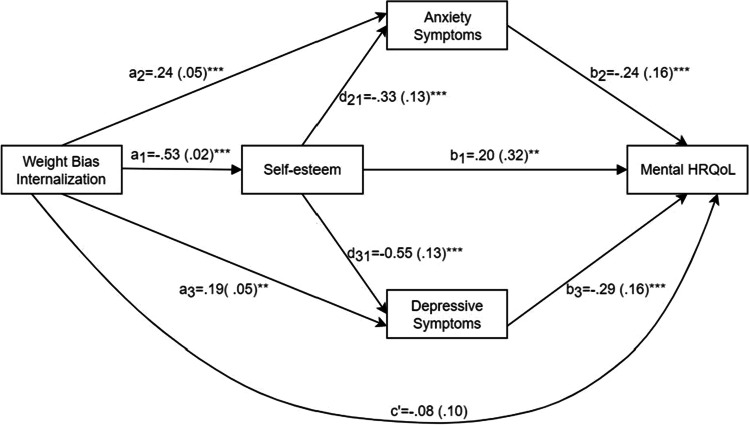
Standardized path coefficients and standard errors of the PROCESS path model among weight bias internalization, self-esteem, anxiety symptoms, depressive symptoms, and mental HRQoL. **P < 0.01, ***P < 0.001

The standardized path coefficients and standard errors (SEs) of the path model, with scores for mental HRQoL as the dependent variable, are presented in Fig. 2, showing that all path coefficients were significant, except for the direct association between WBI and mental HRQoL. The total effect of WBI on mental HRQoL was significant (*β* =  − 0.76, 95%CI = [− 0.97, − 0.56]). The direct effect of WBI on mental HRQoL was not significant. Table 4 shows the standardized coefficients and CIs for the total and individual indirect effects and the model statistics. The results showed that the mediating roles of self-esteem (*β* =  − 0.11, 95%CI = [− 0.18, − 0.04]), anxiety symptoms (*β* =  − 0.06, 95%CI = [− 0.11, − 0.02]), and depressive symptoms (*β* =  − 0.05, 95%CI = [− 0.11, − 0.02]) were all significant. Additionally, the chain-mediating effects of WBI → self-esteem → anxiety symptoms → mental HRQoL and WBI → self-esteem → depressive symptoms → mental HRQoL were significant.

**Table 4:** Mediating effect of self-esteem, anxiety symptoms, and depressive symptoms in the association between WBI and mental HRQoL, controlling for gender, age, and BMI. Standardized coefficients reported

Effect	*β* (SE)	95%CI	Proportion	*n*	Model *R*^2^	*F* (*df*)
Total indirect effect	− 0.34 (0.04)	[− 0.43, − 0.26]	100.00%	246	0.50	33.88 (7,238)***
WBI → self-esteem → mental HRQoL	− 0.11 (0.04)	[− 0.18, − 0.04]	30.70%			
WBI → anxiety symptoms → mental HRQoL	− 0.06 (0.02)	[− 0.11, − 0.02]	16.59%			
WBI → depressive symptoms → mental HRQoL	− 0.05 (0.02)	[− 0.11, − 0.02]	15.80%			
WBI → self-esteem → anxiety symptoms → mental HRQoL	− 0.04 (0.02)	[− 0.08, − 0.02]	12.46%			
WBI → self-esteem → depressive symptoms → mental HRQoL	− 0.08 (0.02)	[− 0.14, − 0.04]	24.42%			

*WBI*, weight bias internalization; *HRQoL*, health-related quality of life. ***P < 0.001

## Discussion

The prevalence of psychopathology in bariatric surgery patients is concerning because it results in poor QoL and health outcomes, and may persist after surgery [27, 28]. Therefore, there is a need to explore potential risk factors and mechanisms of action related to psychopathology in these patients. Although WBI is common in populations with obesity, little consideration has been given to the role of WBI in contributing to psychopathologies. This study investigated the status of WBI and HRQoL in pre-bariatric surgery patients. We validated our hypothesis that WBI is negatively correlated with physical and mental HRQoL through low self-esteem and increased psychological distress. These findings extend the existing research on WBI and QoL, and have significant clinical implications for screening preoperative psychopathology and optimizing surgical outcomes in populations with obesity.

Self-esteem mediated the effects of WBI on HRQoL (i.e., in models with either physical or mental HRQoL as the dependent variable). As a positive psychological variable, self-esteem in pre-bariatric patients with obesity could affect the adverse effects of WBI. WBI causes individuals to impose negative, external, and weight-related stereotypes on themselves, leading to perceived stress and self-deprecation. Self-esteem allows individuals to adjust their coping strategies to cushion the impact of perceiving the pressure of a threat [29]. Meanwhile, low self-esteem disrupts the endocrine system [30], damaging health and making individuals more vulnerable to negative mood states [31]. Furthermore, WBI can also directly affect individuals’ emotional states: the higher the WBI levels, the more serious their anxiety and depressive symptoms, implying psychological distress; such distress, in turn, predicts greater perceived impairment in HRQoL [32]. These results support emerging evidence that WBI may be linked to a greater severity of psychopathology than negative self-evaluation [33, 34].

There were unique paths in the association between WBI and the different domains of HRQoL in this study. In addition to validating the important role played by psychopathology in the above associations, an interesting finding of the current study was that depressive symptoms were not related to physical HRQoL, which is inconsistent with previous studies [35]. This may be due to the use of different measurement tools. We used self-reported measures of depressive symptoms, which may have yielded milder results than those of formal clinical screening for depression. Another hypothesis to account for this finding is that depressive symptoms are associated with particular subdomains of HRQoL. Indeed, the effect sizes of the impact of depressive symptoms on mental HRQoL were larger than those on physical HRQoL [36]. Further research is required to explore the relationship between depressive symptoms and physiological health.

Sociodemographically, our results suggest that WBI, anxiety, and depressive symptoms are correlated with gender, but not with BMI; these results are consistent with those of prior research [37, 38]. Specifically, the female population in our sample reported more mental health problems. This might be due to the social focus on weight and the idea of thinness as beauty, as women are more vulnerable to weight-related stigma and tend to have lower perceptions of body image than men [38]. Studies have found that regardless of weight (i.e., normal weight, overweight, or obesity), women are more concerned about their body image than men [39], which leads to more negative psychological profiles [40]. These findings emphasize the importance of paying more attention to the mental health of bariatric surgery candidates, especially female patients, and aggressive interventions to reduce the adverse effects of social bias against people with obesity.

Overall, positive body image refers to one’s love, respect, acceptance, and appreciation of one’s own body [41]. Conversely, self-deprecation and internalized bias toward body image can increase the risk of psychopathology, and decrease the assessment of the overall health status. For individuals with obesity, we recommend considering interventions based on weight bias as part of clinical treatment, with the goal of not only reducing internalized prejudicial attitudes, but also focusing on promoting their self-respect and self-acceptance.

### Theoretical and Clinical Implications

Although existing evidence correlates WBI with greater perceived impairment in HRQoL in overweight and obese individuals, the underlying psychological mechanisms remain unclear [42]. Our results extend the current findings that negative psychological profiles, including low self-esteem, increased anxiety, and depression, have important mediating effects on these relationships. It has been demonstrated that WBI and the physical and mental health issues reported in pre-bariatric surgery patients may persist in the postoperative period, or even affect weight loss outcomes [43]. Therefore, this study supports the implementation of interventions designed to reduce WBI as an important topic in the clinical treatment of obesity. Accordingly, longitudinal designs are warranted to further evaluate the effectiveness of these interventions in improving physical and psychological health status, and optimizing surgical outcomes in patients with obesity.

### Limitations and Future Research Prospects

First, although the present study provides the necessary evidence for the purported psychological mechanisms, the cross-sectional design is insufficient to determine causality. We modeled the theoretical structure based on existing findings and relevant theoretical models that state that internalizing weight bias leads to poorer health status and quality of life [19, 33, 42, 44]. However, to our knowledge, the prior longitudinal evidence required to establish the directionality of this relationship is lacking. Furthermore, prospective studies have found that thin-ideal internalization and body dissatisfaction predict subsequent increases in depressive symptoms [18]. It has also been reported that depression longitudinally predicts poorer QoL and physical illness [32]. However, due to the overall lack of longitudinal studies on this topic, the causal relationships between WBI, self-esteem, depression, and anxiety are currently unclear. Future longitudinal and experimental approaches are necessary to examine the temporal sequence of the internalized weight bias-physical and mental health problem relationship, and the psychological mediators of this association over time to elucidate the causal processes involved.

In addition, this study excluded a subset of patients who were unable to complete the questionnaire independently, including those with serious mental health problems requiring referral for psychiatric treatment; based on prior research, this may reduce the association between WBI and adverse psychosocial problems [45].

Another major limitation is the lack of diversity in the assessment of WBI. We relied on the subjective assessment tool of WBIS, which reflects the self-perceived WBI of patients, but not the actual discrimination they experienced. Future studies could combine the objective and subjective assessments of weight-related discrimination experienced by patients to provide a more comprehensive analysis of the negative impact of WBI on patients with obesity.

## Conclusion

In addition to confirming the pre-existing finding that WBI is negatively associated with physical and mental HRQoL, our study also revealed significant mediating effects of self-esteem, anxiety, and depressive symptoms on this association in pre-bariatric surgery patients. Our study supports that interventions targeting the reduction of WBI and improvement of self-esteem may be clinically important in the medical care of patients with obesity. Accordingly, longitudinal research is warranted to evaluate the effectiveness of these interventions in this population further.
